# Longitudinal stability of a multimodal visco-elastic polyacrylamide gel phantom for magnetic resonance and ultrasound shear-wave elastography

**DOI:** 10.1371/journal.pone.0250667

**Published:** 2021-05-21

**Authors:** Masashi Usumura, Riwa Kishimoto, Koki Ishii, Eika Hotta, Jeff Kershaw, Tatsuya Higashi, Takayuki Obata, Mikio Suga

**Affiliations:** 1 Department of Medical Engineering, Graduate School of Science and Engineering, Chiba University, Chiba, Japan; 2 QST Hospital, National Institutes for Quantum and Radiological Science and Technology, Chiba, Japan; 3 Applied MRI Research, National Institute of Radiological Sciences, National Institutes for Quantum and Radiological Science and Technology, Chiba, Japan; 4 Department of Molecular Imaging and Theranostics, National Institute of Radiological Sciences, National Institutes for Quantum and Radiological Science and Technology, Chiba, Japan; 5 Center for Frontier Medical Engineering, Chiba University, Chiba, Japan; University of Montreal, CANADA

## Abstract

We evaluated the long-term stability of a newly developed viscoelastic phantom made of polyacrylamide (PAAm) gel for magnetic resonance elastography (MRE) and ultrasound-based shear-wave elastography (US SWE). The stiffness of the cylindrical phantom was measured at 0, 13 and 18 months. Storage and loss moduli were measured with MRE, and shear-wave speed (SWS) was measured with US SWE. Long-term stability was evaluated in accordance with the Quantitative Imaging Biomarker Alliance (QIBA) profiles for each modality. The initial storage and loss moduli of the phantom were 5.01±0.22 and 1.11±0.15 respectively, and SWS was 2.57±0.04 m/s. The weight of the phantom decreased by 0.6% over the 18 months. When measured with MRE, the stiffness of the phantom decreased and changes to the storage and loss moduli were -3.0% and -4.6% between 0 and 13 months, and -4.3% and 0.0% between 0 and 18 months. The US measurements found that SWS decreased by 2.4% over the first 13 months and 3.6% at 18 months. These changes were smaller than the tolerances specified in the QIBA profiles, so the viscoelastic PAAm gel phantom fulfilled the condition for long-term stability. This new phantom has the potential to be used as a quality assurance and quality control phantom for MRE and US SWE.

## Introduction

Elastography is a non-invasive imaging technique that is expected to offer new quantitative biomarkers for the evaluation of tissue stiffness and the diagnosis of disease such as liver fibrosis or tumor malignancies [1–4]. Commercial magnetic resonance (MR)-imaging and ultrasound (US) elastographic systems are available for clinical use [4–6]. MR elastography (MRE) consists of three steps: i) vibrating the subject, ii) acquiring wave images using an MRE pulse sequence with a motion-encoding gradient (MEG), and iii) processing the wave images to calculate the stiffness. In shear-wave elastography (SWE), which is one of several types of US elastography, an acoustic radiation force impulse (ARFI) is used to generate shear waves in the sample, and the shear wave motion is tracked using detection pulses transmitted over a period of time. The shear wave speed (SWS) is then estimated from the shear wave motion at several spatial locations [6]. Based on the fact that SWS is proportional to the square root of the shear modulus when the loss modulus is zero [7], some studies have been performed to test the correlation between SWS and stiffness measured with MRE [5, 7, 8].

The importance of quantitative imaging biomarkers has long been recognized, and efforts to improve the value and practicality of those biomarkers by reducing variability across devices, patients and time are ongoing [9]. The Quantitative Imaging Biomarker Alliance (QIBA), founded by the Radiological Society of North America, has formed committees dedicated to standardizing MRE and SWS measurement through identifying bias in measurements and establishing a phantom suitable for characterization of data acquired from different systems [10, 11]. However, there is no commercially available visco-elastic phantom that is suitable for both MRE and US SWE. Although commercially produced Zerdine phantoms have been used to evaluate US SWE [12, 13], they are not suitable for MRE because the water content is too small to provide sufficient signal. In previous studies, phantoms made of agar gels and gelatin gels were used [14, 15]. However, because the hydrogen and ionic bonds maintaining crosslinking of the polymer chain are not strong, phantoms made of agar or gelatin gels are not stable in the long term [15]. For a phantom to be suitable as a standard, longitudinal change in its stiffness must be small.

In an effort to obtain higher stability, we have developed a phantom made of polyacrylamide (PAAm) gel that can be measured with both MRE and US SWE [16]. The PAAm gel is composed of a three-dimensional network polymer and a large amount of liquid, which is the source of the MR signal. The storage modulus (G’) of the PAAm gel is mainly dependent on the concentration of the acrylamide, while the density of the three-dimensional network polymer is mainly dependent on the concentration of the cross-linker [17]. On the other hand, the loss modulus (G”) is mainly dependent on the ratio of glycerin to water [18].

The purpose of this study was to evaluate the long-term stability of visco-elasticity for a phantom made of PAAm gel, and to evaluate whether such a phantom meets the requirements to be a standard phantom for MRE and US SWE.

## Materials and methods

### Phantom

We made a cylindrical phantom (diameter = 11 cm, height = 10 cm) using PAAm gel. Aluminum oxide powder was added to the PAAm gel to generate US scattering, and glycerin was added to a PAAm gel solvent to adjust the viscosity. To make the phantom, 12 wt% acrylamide (Acrylamide (monomer), 00809–85, Nacalai Tesque, Inc.), 45 wt% glycerin (Glycerol, 075–00611, FUJIFILM Wako Pure Chemical Corporation), 42 wt% water (Distilled Water, 041–16786, FUJIFILM Wako Pure Chemical Corporation), 1 wt% aluminum oxide powder, and a total of less than 0.4 wt% crosslinker, polymerization accelerator, and polymerization initiator was used. The procedure for making the phantom is as follows. First, degas while mixing acrylamide, glycerin, and crosslinker in water. Next, chill this mixture to 6 degrees, mix in the aluminum oxide powder and polymerization initiator, and finally add the polymerization accelerator. The weight of the phantom was measured immediately after construction (0 months) and after the third examination (18 months). In a MRE study using a vibration frequency of 62.5 Hz [16], the tan δ (= G” / G’) of the liver was reported to be approximately 0.3 for both healthy volunteers and patients with liver fibrosis [19]. The relative concentration of the base components in the gel were adjusted to match that value of tan δ. The phantom was wrapped in plastic film (Asahi Kasei Corp., Tokyo, Japan) to prevent drying, and stored in a black sealed polypropylene container (diameter = 15.2 cm, height = 13.7 cm, DIC PLASTICS Inc., Saitama, Japan) at room temperature.

### MR elastography

MR examination was performed using a MAGNETOM Skyra (Siemens Healthcare, Erlangen, Germany) with an 18 channel body matrix coil and a custom-made cylindrical passive pneumatic driver connected to a commercial loudspeaker, based on a design used in a previous study [20]. The passive driver was positioned at the center of the top surface of the phantom. A spin-echo echo-planar MRE sequence (work in progress) was used to acquire axial wave images. Imaging parameters were as follows: MEG frequency (continuous sinusoidal vibration) = 62.5 Hz, repetition time/echo time = 2400/97 ms, voxel size = 3.0×3.0×3.0 mm^3^, imaging matrix = 128×128, and field of view = 384×384 mm^2^. Room temperature was maintained at approximately 21°C.

**Fig 1:**
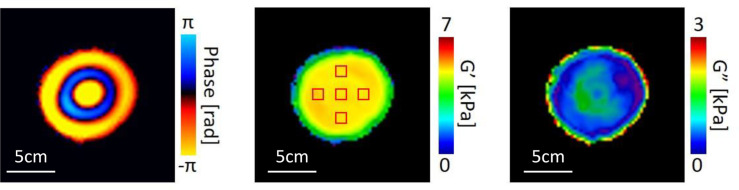
Magnetic Resonance Elastography (MRE) images. Left: Wave image, Center: Storage modulus (G’) map, Right: Loss modulus (G”) map. The five squares on the storage modulus map indicate ROIs.

Fig 1 shows the MRE images for the phantom. G’ and G” were calculated with a three-dimensional integral-type reconstruction formula (ITRF) [21]. Using the Voigt model for viscoelasticity, the SWS for MRE [SWSmre (m/s)] was calculated from G’ and G" using the equation [22].

$$SWSmre=\sqrt{\frac{2({G\prime }^{2}+{G\prime \prime }^{2})}{\rho (G^{\prime }+{\left({G^{\prime }}^{2}+{G^{\prime \prime }}^{2}\right)}^{\frac{1}{2}})}}$$

Here, *ρ* is the density of the material. Five regions of interest (ROIs) of size = 12 × 12 mm^2^ were drawn at a depth of 50 mm inside the phantom so as to avoid the peripheral area and hence any error due to edge effects (Fig 1).

MR examination was performed at 0, 13 and 18 months. The measurement was performed only once for the first and second examinations, but for the third examination at 18 months the same measurement was repeated five times with re-setting of the pneumatic driver and the coil in order to evaluate the reproducibility of measurement. The mean value and standard deviation (SD) across ROIs were calculated for each examination.

### US shear wave elastography

**Fig 2:**
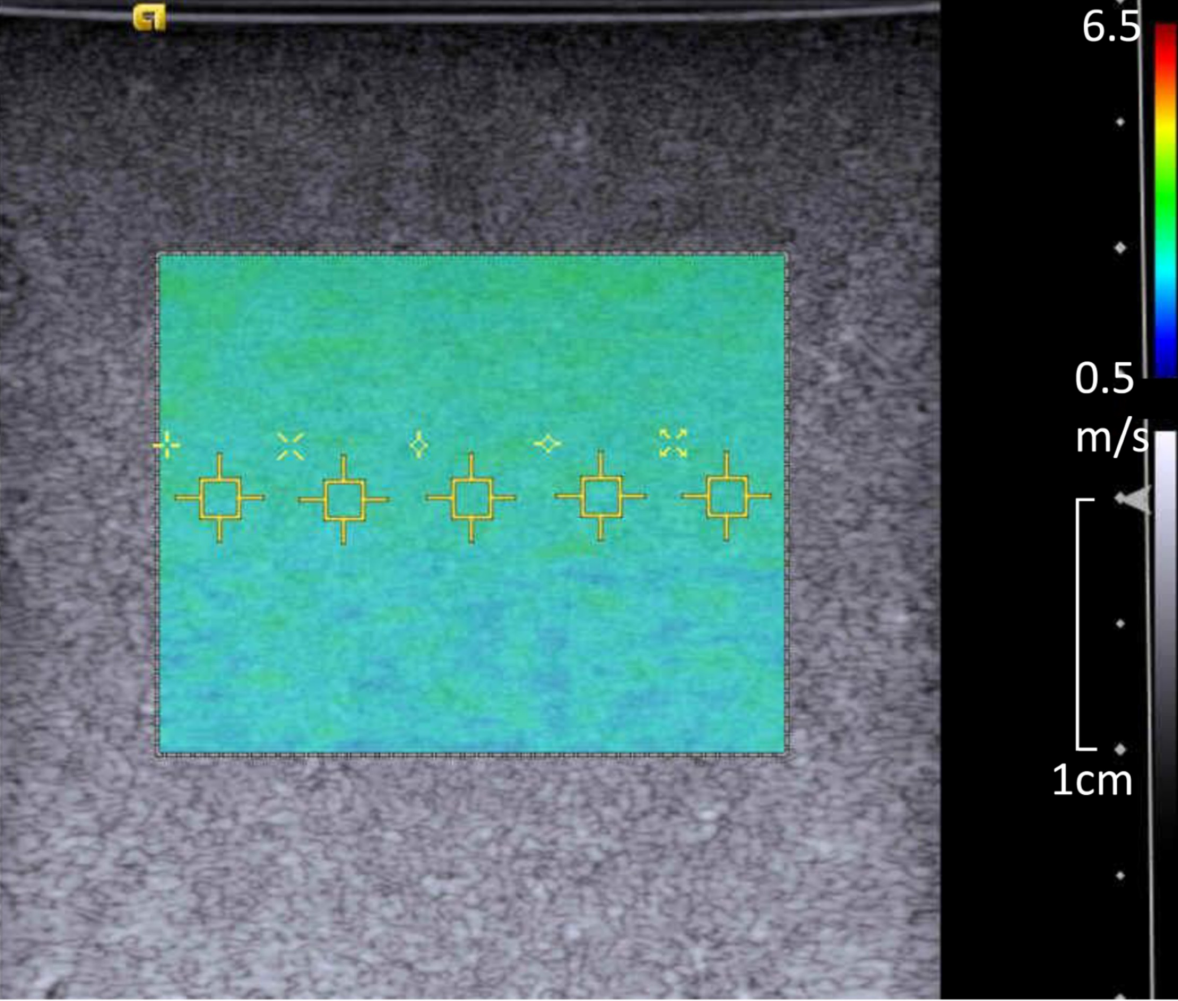
2D color-coded shear wave elastography (2D SWE) image. The color box corresponds to the 2D-SWS distribution and the small yellow squares indicate ROIs for SWS measurement. Five ROIs were placed at a depth of 20 mm.

US SWE was performed using the ACUSON S3000 ultrasound system (Siemens Medical Solution, Mountain View, USA) with a linear probe (9L4) held on top of the phantom by a retort clamp. SWS measurement was performed with two-dimensional color-coded SWE (2D SWE) utilizing ARFI technology. SWS was also measured at 0, 13 and 18 months at the same time as the MRE was performed. For the first and second examinations, five ROIs were set at a depth of 20 mm in the phantom (Fig 2), and measurements were repeated three times without re-setting the probe. For the third examination, the same procedure was repeated five times after re-setting the probe to evaluate the reproducibility. The mean value and SD were calculated for each examination from 15 SWS (5 ROIs x 3 repetitions) estimates. The imaging parameters were as follows: ROI size = 1.5 × 1.5 mm^2^ (fixed and unchangeable), push pulse frequency = 5.7 MHz, detect pulse frequency = 6.0 MHz. The temperature of the room where US was performed was also maintained at 21°C.

### Statistical analysis

To evaluate the statistical significance of any differences between the examinations at 0, 13 and 18 months, either one-way analysis of variance (ANOVA) or the Kruskal-Wallis test was used after the Shapiro-Wilk test for normality. In the QIBA profiles “MR elastography of the liver” [10] and “Ultrasound measurement of SWS for estimation of liver fibrosis” [11], the % change in measurement is defined as 2 × ⎜E1-E0⎜/ (E1+E0) x 100, where E1 is the mean stiffness obtained from the current measurement, and E0 is the value obtained from the previous measurement. This quantity was calculated for the G’, G” and SWS estimates.

The reproducibility after re-setting the pneumatic driver, coil and probe was evaluated using the data of the third examination. The coefficient of variation (CV = SD/mean) was calculated from the five measurements for both MRE and US SWE.

Statistical analysis was carried out using the SPSS 25.0 software package (SPSS Inc, Chicago, IL), with a p-value < 0.05 considered statistically significant.

## Results

### Longitudinal change of the weight, viscoelasticity and SWS

Longitudinal changes in the phantom are summarized in Table 1. The weight of the phantom was 1115.3 g at the first examination and 1108.5 g at the third examination 18 months later. The percent change in weight was a 0.6% decrease over 18 months.

**Table 1:** Longitudinal changes to the characteristics of the phantom.

	0 month	11 months	18 months
**Weight (g)**	1115.3		1108.5
**G’ (kPa)**	5.01 ± 0.22	4.86 ± 0.10	4.80 ± 0.06
**G" (kPa)**	1.11 ± 0.15	1.07 ± 0.11	1.11 ± 0.13
**tanδ**	0.22 ± 0.03	0.22 ± 0.02	0.23 ± 0.03
**SWSmre (m/s)**	2.28 ± 0.05	2.24 ± 0.02	2.24 ± 0.02
**SWS (m/s)**	2.57 ± 0.04	2.51 ± 0.03	2.48 ± 0.05

G’: Storage modulus, G”: Loss modulus, tanδ: G”/G’, SWSmre: Shear wave speed calculated from G’ and G”, SWS: Shear wave speed obtained using ultrasound.

**Fig 3:**
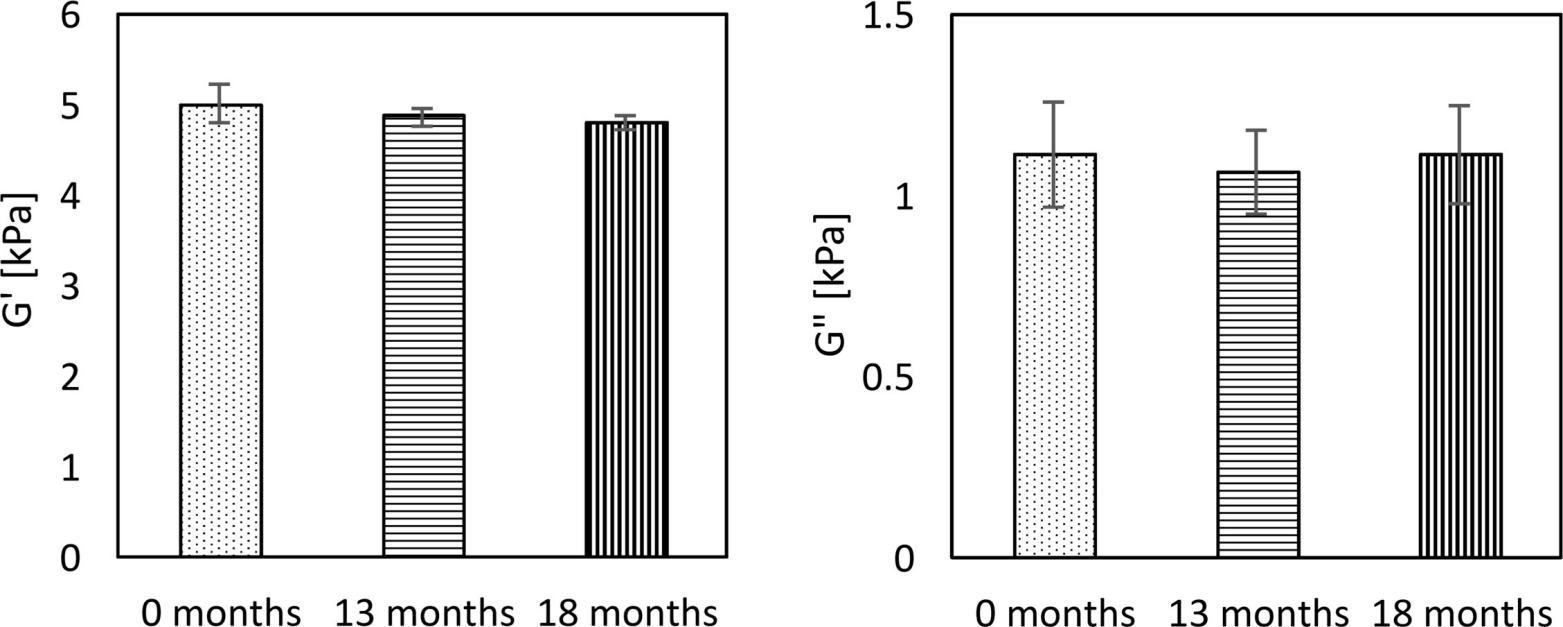
Longitudinal change of the storage modulus (G’) (a) and the loss modulus (G”) (b) obtained from MRE. The error bars represent the SD of five ROIs. There were no statistical differences between G’ and G” over any of the time periods.

**Fig 4:**
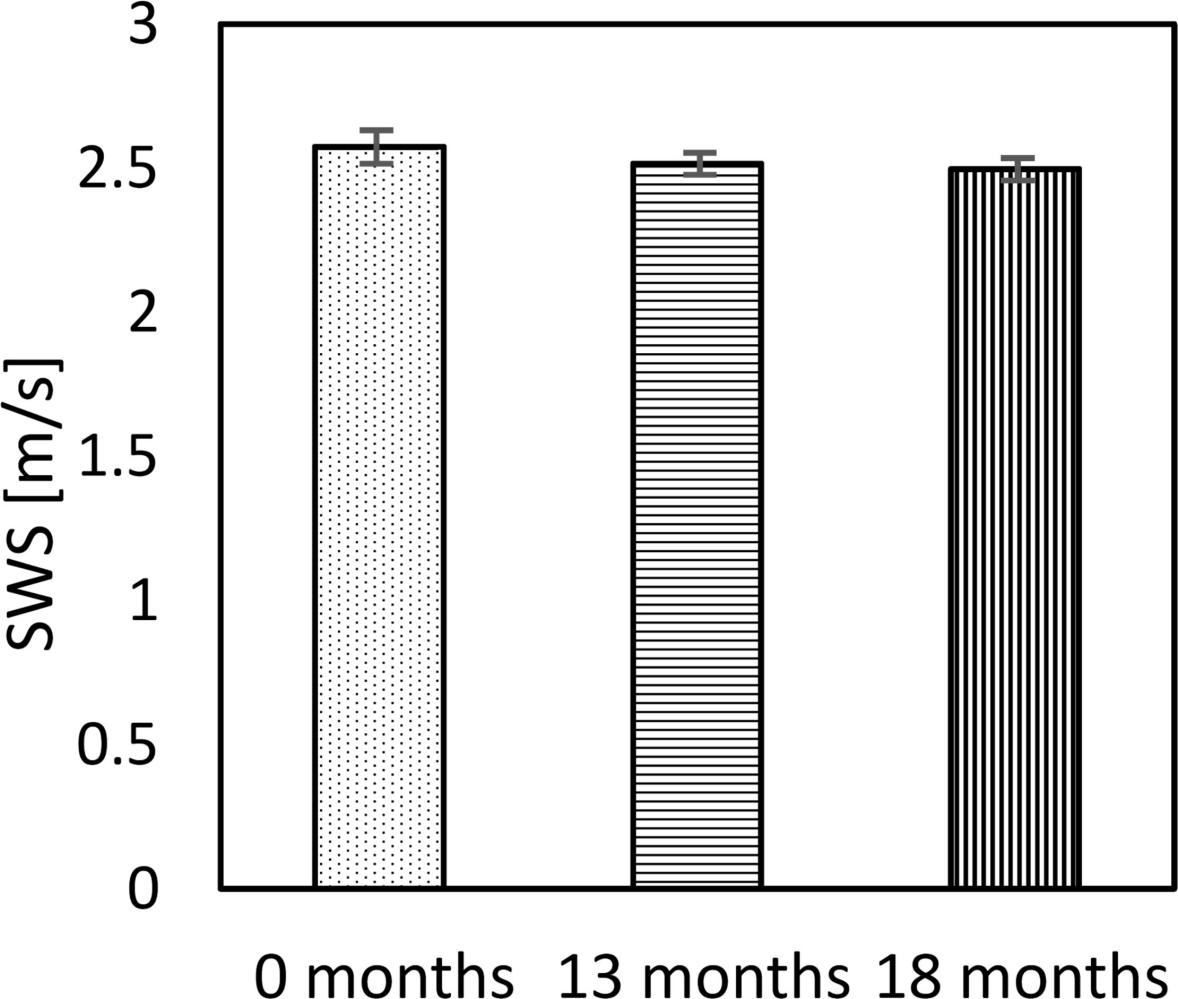
Longitudinal change of SWS obtained from US SWE. The error bars represent the SD of 15 SWS (5 ROIs x 3 times) estimates. A Kruskal-Wallis test found a significant difference between the SWS at each time point (p = 0.00).

At the first examination, G’ and G” obtained from MRE were 5.01±0.22 kPa and 1.11±0.15 kPa, respectively, with tan δ being 0.22±0.03, and the SWS from US SWE was 2.57±0.04 m/s. Longitudinal change in G’ and G” is shown in Fig 3 and that of SWS is in Fig 4. The % change in G’ and G” was -3.0% and -4.6%, respectively, between the first and second measurements, and -4.3% and 0.0% over the full 18 months (Fig 3). Testing with ANOVA found no statistical differences in G’ (p = 0.09) and G” (p = 0.81) over the 18-month period. SWS decreased 2.4% in the first 13 months and 3.6% for the full 18 months. A Kruskal-Wallis test found a significant difference between the measurements over the 18 months (p = 0.00) (Fig 4).

**Fig 5:**
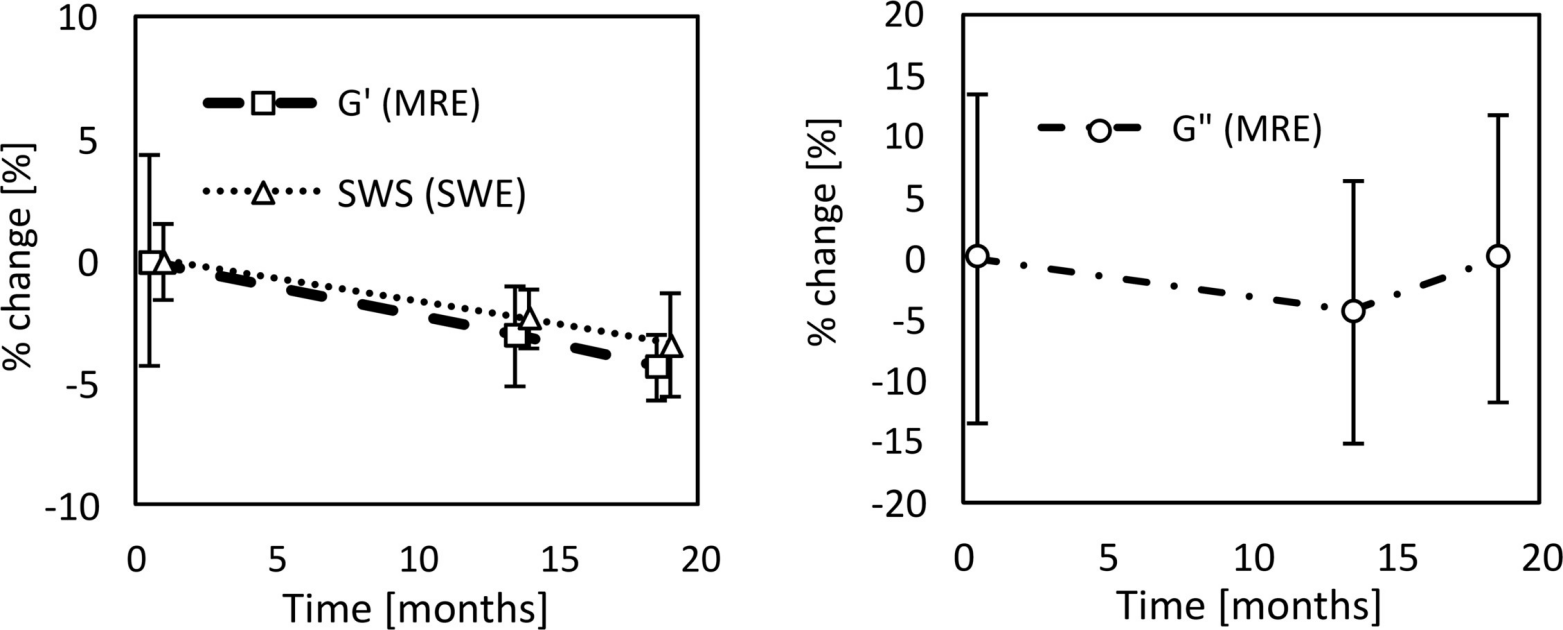
The % change in the storage modulus (G’) and SWS (a) and the loss modulus (G”) (b). Note that the scale of the y-axis in (b) is twice that in (a). Each error bar represents the coefficient of variation (CV). G’ and SWS decreased with a similar trend. G” did not show a clear trend due to the large CV.

Fig 5 plots the % change of G’, G” and SWS as a function of time. Each error bar represents the corresponding CV. G’ and SWS decreased gradually with a similar trend. On the other hand, there is no clear trend for G” because of the large CV.

### Inter-measurement reproducibility after re-setting at the third examination

G’ and G” were 4.83±0.04 kPa and 1.13±0.04 kPa, respectively, and the CVs of five separate measurements were 0.83% and 3.39%, respectively. SWS was 2.48±0.01 m/s with a CV of 0.40%.

## Discussion

The conditions for a suitable US SWE phantom are defined in the recently released QIBA US-SWS profile “Ultrasound measurement of SWS for estimation of liver fibrosis” [11]. Although the profile recommends that testing to verify the characterization of a phantom is performed by the QIBA committee using a rented Verasonics US system [11], that information was unavailable at the time our examinations were performed. Alternatively, we measured the stiffness with both MRE and US SWE. As shown in Fig 5, G’ and SWS decreased in a similar manner over 18 months, whereas G” did not show a clear trend due to the large variability. Even though the SD of the G” measurements is about the same as that for G’, the CV of G” is much larger because of the lower mean values. The fact that G’ and SWS, which are obtained with different modalities, demonstrated a similar trend over time is thought to support the accuracy of the measurements. Despite the similarity of these trends, the change of G’ was not significant whereas that for SWS was. The smaller SD of the SWS is probably due to the larger number of SWS measurements.

When comparing US SWE and MRE, the SWS obtained with ultrasound was about 15% higher than SWSmre. This can be attributed to the difference in the frequency range where measurements were made. The frequency of the MEG for MRE was 62.5 kHz, while US SWE was sensitive to shear waves with the range 100–500 kHz, and it is reported that the storage and loss modulus vary depending on frequency [19].

It is stated in the QIBA profile for US-SWS that the phantom should be re-weighed at six-month intervals after initial delivery, and if the phantom weight changes by more than ±0.5% the phantom should be re-characterized prior to further use. Since we did not measure the weight of our phantom at 6 months and the weight loss was 0.6% at 18 months, strictly speaking our phantom did not fulfill the criterion above and re-certification was probably required. However, with regards to the stability of the acoustic properties, the QIBA profile states that the phantom can continue to be used if the change in SWS is less than ±5% over 6 months. The SWS of our phantom decreased by 2.4% in 13 months and 3.6% over 18 months, which means that it meets the requirement for SWS stability despite the weight change. For MRE, the % change in G’ and G” was -3.0% and -4.6%, respectively, between the first and second measurements, and -4.3% and 0.0% over the full 18 months, which are also less than ±5% and fulfill the QIBA condition.

In a previous study that measured the weight and elastic modulus of phantoms made of various materials over a nine-month period, it was reported that the elastic modulus increased with weight loss [15]. In that case it was thought that the weight loss was due to drying that caused the phantom to harden. In our study, the phantom lost weight but the storage modulus and SWS, representing stiffness, also decreased. It is suspected that material degradation due to weakening of the cross-linkage in the PAAm gel over time made the phantom softer.

Before evaluating the long-term stability of the phantom, it was necessary to verify the reproducibility of the measurement [23]. After five measurements with re-setting, the CV of the mean value was less than 1% for G’ and SWS, and less than 5% for G”. This suggests very high reproducibility of measurement, especially for G’ and SWS.

A linear probe was used for this study because two-dimensional color-coded SWE (2D SWE) is unavailable on the Acuson S3000 system when using a convex probe. With a linear probe, it was possible to measure the SWS after confirming the homogeneity of the phantom on the 2D SWE images. Although a convex probe is usually used to measure liver stiffness, we found in a previous study that there was no substantial difference in SWS measurement between a linear and a convex probe when the stiffness of the phantom is around 5 kPa [7, 23]. It is not thought that the difference between the probes is important in this study.

Even though antennas used in MRE can heat up during MRI scanning and warm up the phantom, we did not monitor the temperature of the phantom in this study. In a separate experiment, we have monitored the temperature of a phantom during MR scanning, and found that the temperature rose less than 1°C even after 7 hours of continuous imaging using a sequence with relatively high SAR (specific absorption rate) [24]. Furthermore, the air inside the gantry is circulated to prevent the temperature from rising. It is unlikely that variation in the temperature of the phantom had a substantial effect on the results.

The phantom was designed to have a stiffness higher than normal liver to simulate pathological tissue (e.g. cirrhosis), as well as other organs that may be examined, such as spleen, kidney, prostate, and thyroid. In addition, a stiffer phantom has the advantage of stabilizing the measurement because it is less likely to be distorted by its own weight or be deformed by placing a passive driver or probe on top of it.

There are some limitations in our current study. The ultrasound attenuation and speed of sound in our phantom were not measured so it is uncertain whether this phantom fulfills all of the specifications defined in the QIBA profile for US measurement of SWS [11]. As noted earlier, the profile had not been released at the time we developed this phantom. A new phantom that satisfies the QIBA specifications has since been created [16] and the longitudinal change to the characteristics of the phantom after 28 months are included as S1 Data. Although measurements were not made at 6 months intervals, changes to the SWS and SWSmre were less than 10%. Assuming that the process of change was constant over the 28 months, the change to the stiffness at 6 months may be estimated to be less than 5%, which meets the QIBA criteria. Examination of more phantoms with different stiffness would help reinforce our results.

In conclusion, a visco-elastic phantom made of PAAm gel fulfilled the condition for long-term stability of stiffness and SWS specified in the QIBA profile. Visco-elastic phantoms made of PAAm gel have the potential to be used for quality assurance and quality control for MRE and US SWE.

## Supporting information

S1 Data Longitudinal changes to the characteristics of a phantom that satisfies the QIBA specifications. (DOCX)
