# Supplementary material for: Longitudinal stability of a multimodal visco-elastic polyacrylamide gel phantom for magnetic resonance and ultrasound shear-wave elastography
Source: PLoS One. 2021 May 21;16(5):e0250667. doi: 10.1371/journal.pone.0250667 (PMC8139483; doi:10.1371/journal.pone.0250667)
Supplement: S1 Data — (DOCX) [file pone.0250667.s001.docx]

S1 Data

**Longitudinal changes to the characteristics of a phantom that satisfies the QIBA specifications.**

|  | 0 month | 28 months |
| --- | --- | --- |
| Weight (g) |  | 1006.3 |
| G' (kPa) | 2.98 ± 0.06 | 3.16 ± 0.28 |
| G" (kPa) | 0.80 ± 0.05 | 0.84 ± 0.10 |
| tanδ | 0.27 ± 0.02 | 0.27 ± 0.03 |
| SWSmre (m/s) | 1.77 ± 0.02 | 1.82 ± 0.08 |
| SWS (m/s) | 2.19 ± 0.05 | 2.01 ± 0.08 |

G’: storage modulus, G”: loss modulus, tanδ: G”/G’, SWSmre: shear wave speed calculated from G’ and G”, SWS: shear wave speed obtained using ultrasound
